# Evaluation of Enzymatic Cleaning on Food Processing Installations and Food Products Bacterial Microflora

**DOI:** 10.3389/fmicb.2020.01827

**Published:** 2020-08-11

**Authors:** Laurent Delhalle, Bernard Taminiau, Sebastien Fastrez, Abdoulaye Fall, Marina Ballesteros, Sophie Burteau, Georges Daube

**Affiliations:** ^1^Fundamental and Applied Research for Animals and Health, Department of Food Science, University of Liège, Liège, Belgium; ^2^Realco SA, Ottignies-Louvain-la-Neuve, Belgium; ^3^Genalyse Partner SA, Herstal, Belgium

**Keywords:** enzyme, cleaning, food, microflora, spoilage, contamination, metagenetics

## Abstract

Biofilms are a permanent source of contamination in food industries and could harbor various types of microorganisms, such as spoiling bacteria. New strategies, such as enzymatic cleaning, have been proposed to eradicate them. The purpose of this study was to evaluate the impact of enzymatic cleaning on the microbial flora of installations in a processing food industry and of the final food product throughout its shelf life. A total of 189 samples were analyzed by classical microbiology and 16S rDNA metagenetics, including surface samples, cleaning-in-place (CIP) systems, and food products (at D_0_, D_end of the shelf life_, and D_end of the shelf life +7 days_). Some surfaces were highly contaminated with spoiling bacteria during conventional cleaning while the concentration of the total flora decreased during enzymatic cleaning. Although the closed circuits were cleaned with conventional cleaning before enzymatic cleaning, there was a significant release of microorganisms from some parts of the installations during enzymatic treatment. A significant difference in the total flora in the food products at the beginning of the shelf life was observed during enzymatic cleaning compared to the conventional cleaning, with a reduction of up to 2 log CFU/g. Metagenetic analysis of the food samples at the end of their shelf life showed significant differences in bacterial flora between conventional and enzymatic cleaning, with a decrease of spoiling bacteria (*Leuconostoc* sp.). Enzymatic cleaning has improved the hygiene of the food processing instillations and the microbial quality of the food throughout the shelf life. Although enzymatic cleaning is not yet commonly used in the food industry, it should be considered in combination with conventional sanitizing methods to improve plant hygiene.

## Introduction

Biofilms are multicellular communities held together by a self-produced, extra polymeric substance (EPS). The mechanisms that different bacteria employ to form biofilms vary, depending on environmental conditions and specific strain attributes (López et al., 2010). Several studies have demonstrated the presence of biofilms in various food industries, such as breweries, dairies, fresh vegetables industries, poultry and meat cutting plant (Marchand et al., 2012; Giaouris et al., 2014; Kim et al., 2017; Adator et al., 2018; Parijs and Steenackers, 2018). Biofilms are a source of microbial contamination leading to food spoilage and shelf life reduction and a potential way of pathogen transmission (Wirtanen and Salo, 2016; Giaouris and Simões, 2018). In particular, approximately 60% of food-borne infections results from microbial transfer from equipment surfaces to processed foods (Bridier et al., 2015). Product-contact surfaces in the food process may contaminate the product directly, i.e., the product touching over the surface will potentially lead to microbial contamination (Gibson et al., 1999).

Bacteria embedded in a biofilm are 100–1000 times more resistant to cleaning and sanitizing chemicals than the corresponding planktonic cells (Gilbert et al., 2002). Nevertheless, CIP procedures still leave residual microorganisms on equipment surfaces, thus resulting in biofilm formation (Bremer et al., 2006; Kumari and Sarkar, 2016; Parijs and Steenackers, 2018). The time it takes for biofilm to form depends on the frequency of cleaning and disinfection regimes: if there is a long period between cleaning/disinfection treatments, then there is more time for biofilm to form on surfaces (Gibson et al., 1999). Increased biofilm resistance to conventional treatment enhances the need to develop new control strategies (Simões et al., 2010; Coughlan et al., 2016).

New strategies has been proposed to eliminate biofilms, i.e., by using enzymes, phages, and bioregulation (Coughlan et al., 2016). The use of enzyme-based detergents as biocleaners, also known as “green chemicals,” can be a viable option to overcome biofilms in the food industry (Lequette et al., 2010; Stiefel et al., 2016; Fleming and Rumbaugh, 2017). Formulations containing several different enzymes are a successful biofilm control strategy (Coughlan et al., 2016). In industrial environments, numerous microbial species coexist within the same biofilm, thus increasing the biochemical heterogeneity of the matrix. Efficient formulations may therefore be composed of mixtures of enzymes with different substrates to destabilize the EPS, such as proteases, cellulases, polysaccharide depolymerases, alginate lyases, dispersin B, and DNAses (Bridier et al., 2015).

However, studies evaluating the efficacy of enzyme-based detergents have been conducted in labs or pilot plant scale models (Oulahal et al., 2007; Lequette et al., 2010; Lefebvre et al., 2016; Stiefel et al., 2016; Nagaraj et al., 2017). Lab models have their own advantages and their own limitations, but they could never mimic the real conditions that can be encountered in industries. This study evaluates the impact of enzymatic cleaning protocols on the microbial flora of installations in the processed food industry and of the final food product throughout its shelf life. The objective is to assess whether enzymatic cleaning could be considered as an alternative to conventional cleaning in food industries to improve the microbial ecology of food processing surfaces and equipment’s.

## Materials and Methods

### Food Process

**FIGURE 1:**
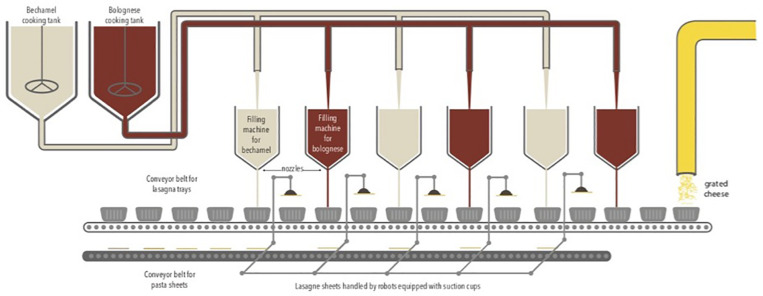
Food production chain of lasagne.

The study was carried out from August to December 2016 in a Belgian food company that produces ready-to-eat lasagne. The production is fully automatic via the successive addition of layers of Bolognese sauce, bechamel sauce, and lasagne sheets. The sauces are pre-cooked and placed successively in a tray via a dosing system. Several filling machines containing sauces are present along the production chain. Lasagne sheets were handled by robots equipped with suction cups. Finally, a layer of grated cheese is placed on top. The food products are packed under a modified atmosphere containing 50% N_2_ and 50% CO_2_. The duration of the production cycle is 48 h continuously with three production cycles per week in a room at 20°C. The production chain is described in Figure 1.

### Cleaning Methods

The basic sequence of the cleaning and disinfection operations (herein, referred together as sanitation) for the open surface and closed circuits is as follows: (1) a pre-rinse with cold water to remove largest residues; (2) cleaning with detergent to remove remaining residues; (3) an intermediate water rinse to remove detergents; (4) disinfection with a chemical agent; and (5) a cold water rinse to remove the disinfectant (Simões et al., 2010; Forsythe and Hayes, 2012). In this company, the installations are cleaned and disinfected three times a week after 48 h of production. For the open surfaces, the installations are cleaned by an alkaline chlorinated solution (EnduroPlus VE6, conc. 3%, Diversey, United States) and by an acid agent (EnduroEco VE9, conc. 3%, Diversey, United States) at 40°C for 15 min per each step. The installations are disinfected by quaternary ammonium (Divosan Extra VT55L, conc. 1%, Diversey, United States) and (peracetic acid Divosan Actif VT5, conc. 1%, Diversey, United States) at room temperature for 15 min each disinfectant. For closed circuits, the pipes are cleaned three times a week with caustic soda (Cipton HD VC151, conc. 3%, Diversey, United States) at 85°C for 90 min by the CIP system. An additional cleaning process is carried out once a week with an acid (Pascal VA5, conc. 1%, Diversey, United States) at 85°C for 15 min, followed by disinfection with peracetic acid (Divosan Actif VT5, conc. 1%, Diversey, United States) at 20°C for 30 min. This cleaning and disinfection protocol of the facilities is referred as “conventional cleaning” in this article.

The enzymatic cleaners were developed by the French National Institute for Research in Agronomy (INRA; Villeneuve d’Ascq, France) and were formulated by a commercial company (Realco, Louvain La Neuve, Belgium). The formulation consists of several enzymes targeting the components of EPS, surfactants, and dispersing and chelating agents (Lequette et al., 2010). Two types of enzymatic cleaning protocols were applied for open surfaces: “reinforced” and “routine” enzymatic cleaning. The reinforced enzymatic cleaning is distinguished from routine enzymatic cleaning by longer treatment duration and higher number and concentration of enzymes. For open surfaces, the installations are cleaned by an alkaline chlorinated solution (EnduroPlus VE6, conc. 3%, Diversey, United States) at 40°C for 15 min, followed by foaming enzymatic solutions at 40°C for 30 min for reinforced enzymatic cleaning (Enzyfoam SG, conc. 3%, Realco, Belgium) and for 15 min for the routine enzymatic cleaning (Bioremfoam, conc. 3%, Realco, Belgium).

The closed circuits are cleaned with enzymatic detergents only once when enzymatic treatment of the facilities is initiated (Biorem A1 + Biorem 10, conc. 0,5% and 0,1%, respectively, Realco, Belgium). The conventional cleaning protocol was applied before using enzymatic detergent at 45°C and pH 7 for 60 min and at pH 9 for 60 min.

For this study, different cleaning treatments were tested over time: conventional cleaning, reinforced enzymatic cleaning, routine enzymatic cleaning 3X/week, 2X/week, and 1X/week, then conventional cleaning again and reinforced enzymatic cleaning. The solutions were applied according to the manufacturer’s recommendations. All factors and the food product remained unchanged, except for the cleaning and disinfection protocol. Table 1 describes the different cleaning and disinfection protocols applied in this study.

**TABLE 1:** Cleaning and disinfection protocols for open surfaces and closed circuits.

ZONE	Open Plant Cleaning (OPC) of the surfaces							Cleaning In Place (CIP) of closed circuits and tanks		
Period (wk)	1–3	4–5	6–7	8–9	10–11	12–13	14–15	1–15		4

Protocol	Conventional cleaning	Enzymatic cleaning				Conventional cleaning 2	Enzymatic cleaning			
					
		Reinforced enzymatic cleaning	Routine enzymatic cleaning				Reinforced enzymatic cleaning 2	Conventional treatment		Reinforced enzymatic cleaning
Frequency	3x/wk (every 48 h)	3x/wk (every 48 h)	3x/wk (every 48 h)	2x/wk with enzymatic cleaning + 1x/wk with conventional cleaning	1x/wk with enzymatic cleaning + 2x/wk with conventional cleaning	3x/wk (every 48 h)	3x/wk (every 48 h)	3x/wk (every 48 h)	1x/wk (before the production week)	In addition to the conventional treatment
Cleaning	Alkaline chlorinated15 min, 40°C	Alkaline chlorinated15 min, 40°C	Alkaline chlorinated15 min, 40°C			Alkaline chlorinated15 min, 40°C	Alkaline chlorinated15 min, 40°C”	Caustic soda90 min, 85°C	Acid agent15 min, 85°C	
	Rinsing	Rinsing	Rinsing			Rinsing	Rinsing	Rinsing	Rinsing	
	Acid agent15 min,40°C	Foaming enzymatic detergent 3% (protease, lipase, amylase, oxydo reductase) 30 min, 40°C	Foaming enzymatic detergent 3% (protease, lipase, amylase)15 min, 40°C			Acid agent15 min, 40°C	Foaming enzymatic detergent 3% (protease, lipase, amylase, oxydo reductase) 30 min, 40°C	Alkaline chlorinated30 min, 85°C		Enzymatic detergent (protease, lipase, amylase, oxydo reductase)pH 7, 60 min, 45°CpH 9, 60 min, 45°C
	Rinsing	Rinsing	Rinsing			Rinsing	Rinsing	Rinsing		
Disinfection	Quaternary ammonium15 min, 40°C					Quaternary ammonium15 min, 40°C				
	Rinsing					Rinsing				
	Peracetic acid15 min, 20°C	Peracetic acid15 min, 20°C	Peracetic acid15 min, 20°C			Peracetic acid15 min, 20°C	Peracetic acid15 min, 20°C	Peracetic acid30 min, 20°C		Peracetic acid30 min, 20°C
	Rinsing	Rinsing	Rinsing			Rinsing	Rinsing	Rinsing		Rinsing

### Sampling Collection

The surface samples were the pasta conveyor belt and the nozzles of the dosing machines for Bolognese and bechamel sauces. Surface sampling was conducted several times per week in accordance with the requirements of ISO 18593 regarding surface sampling techniques (ISO, 2016) with sterile wipes (KW-P8030, Conformat, France). The wipes were placed in a sterile plastic bag (IUL 2456, IUL instruments, Spain) with neutralizing buffer (DifcoTM Neutralizing Buffer, Becton, Dickinson and Company, United States). One liter of cleaning water from the closed circuits was collected during enzymatic cleaning into autoclaved glass bottles (GLS 80, DURAN, Germany). The lasagnes were collected at the end of the food chain production line after sealing the tray under modified atmosphere. The food product was the same throughout the study with the same recipe and same packaging. All the samples were placed in a refrigerated box (4°C) and transferred on the same day to the laboratory for analysis. The food samples were stored in a fridge at 8°C to be analyzed at D_0_, D_end the shelf life_, and D_end of the shelf life + 7 days_. Table 2 describes the samples collected during the study in relation to the cleaning protocol.

**TABLE 2:** Samples analyzed by classical microbiology and metagenetic analyses.

Cleaning method		Samples	n
Conventional cleaning		Ingredients (pasta, bolognese sauce, béchamel sauce, and cheese)	4
		Surfaces	8
		Food products at D_0_, D_end of the shelf life_, and D_end of the shelf+ 7 days_	39
Enzymatic cleaning		Surfaces	18
	Reinforced enzymatic cleaning (OPC + CIP)	Food products at D_0_, D_end of the shelf life_, and D_end of the shelf + 7 days_	12
		Cleaning water during enzymatic cleaning	7
	Routine enzymatic cleaning 3x/wk	Surfaces	10
		Food products at D_0_, D_end of the shelf life_, and D_end of the shelf + 7 days_	12
	Routine enzymatic cleaning 2x/wk	Surfaces	7
		Food products at D_0_, D_end of the shelf life_, and D_end of the shelf + 7 days_	12
	Routine enzymatic cleaning 1x/wk	Surfaces	4
		Food products at D_0_, D_end of the shelf life_, and D_end of the shelf + 7 days_	9
Conventional cleaning 2		Surfaces	9
		Food products at D_0_, D_end of the shelf life_, and D_end of the shelf + 7 days_	12
Enzymatic cleaning	Reinforced enzymatic cleaning 2 (OPC)	Surfaces	18
		Food products at D_0_, D_end of the shelf life_, and D_end of the shelf + 7 days_	9
Total			189

*OPC, open plant cleaning; CIP, cleaning in place; D_0_, food product analyzed at the beginning of the shelf life; D_*end of the shelf life*_, food product analyzed at the end of the shelf life; D_*end of the shelf +* 7 *days*_, food product analyzed seven days after the end of the shelf life.*

### Microbiological Analyses

One laboratory licensed by the Belgian Ministry of Public Health and accredited in accordance with the requirements of the ISO 17,025 standard performed all the microbiological analyses. All samples were stored chilled and were examined within 24 h.

Twenty-five grams (25 g) of food or wipes from surfaces were put in a Stomacher bag with a mesh screen liner (80-μm pore size; ref 80015, BioMérieux, France) under aseptic conditions. Physiological water was automatically added to each bag at 1:10 dilution (Dilumat, BioMérieux, France), and the samples were homogenized for 2 min in a Stomacher (Bagmixer, Interscience, France). From this primary suspension, decimal dilutions in physiological water (8.5 g/L sodium chloride) were prepared for microbiological analysis, and 0.1 mL aliquots of the appropriate dilutions were plated onto media for each analysis in triplicate (Spiral plater, DW Scientific, United Kingdom). For the enzymatic cleaning water from closed circuits, one liter (1 L) water from CIP was filtered through 0.45-μm sterile filters (HABG047S6, Merck, Germany).

The following microbiological analyses were performed:

1. Aerobic colony counts, following the requirements of the modified ISO 4833 standard using PCA (Plate Count Agar, #3544475, Bio-Rad, Marnes-la-Coquette, France) at 22°C and incubation for 72 h;
2. Anaerobic colony counts, following the requirements of the ISO 6222 standard using the Reinforced Clostridial agar (BO0251M, Thermo Fischer Scientific, Waltham, United States) at 22°C and incubated for 24 h under anaerobic conditions.

Aerobic colony count were also assessed for surface samples and for cleaning water from closed circuits.

Aerobic colony count is evaluated using the ISO 4833:2003 standard for which incubation temperature of the plates is performed at 30°C. However several studies used a lower incubation temperature than that indicated in the ISO 4833:2003 standard for the detection of psychrophilic bacteria in foodstuffs (Ercolini et al., 2009; Pothakos et al., 2012, 2015; Ribeiro Júnior et al., 2018; Samapundo et al., 2019). Pothakos et al. (2012) have shown a consistent underestimation of the microbial flora with the total viable counts on plates incubated at 30°C (representing the mesophiles) compared on plates incubated at 22°C (indicating the psychrotrophs) for 86 food samples covering a wide range of foods products.

### ATPmetry

Adenosine triphosphate tests are one of the most commonly used hygiene monitoring indicator to check cleaning effectiveness as they are simple and easy to use and provide immediate results (ICMSF, 2012). The surface samples and cleaning water from closed circuits were tested to measure ATP concentration with a commercial kit (QGA-100C, LuminUltra Technologies SAS, Canada), and the results were expressed as pg cATP/ml.

### 16S rDNA Extraction and High-Throughput Sequencing

Bacterial DNA was extracted from each primary suspension, previously stored at −80°C, using the DNEasy Blood and Tissue kit (QIAGEN, Belgium), following the manufacturer’s recommendations.

The resulting DNA extracts were eluted in DNase/RNase-free water, and their concentration and purity were evaluated using optical density with the NanoDrop 2000/2000c spectrophotometer (Thermo Fisher Scientific, United States) by measuring the ratio of the absorbance at 260 nm and 280 nm (A260/280) and at 260 and 230 nm (A260/230). If the DNA concentration exceeds 200 ng/μl, the DNA is diluted 5-fold to avoid PCR inhibition. DNA samples were stored at – 20°C until 16S rRNA amplicon sequencing. PCR-amplification of the V1-V3 region of the 16S rDNA library preparation was performed with the following primers (with Illumina overhand adapters): forward (5′-GAGAGTTTGATYMTGGCTCAG-3′) and reverse (5′-ACCGCGGCTGCTGGCAC-3′). Each PCR product was purified with the Agencourt AMPure XP beads kit (Beckman Coulter; Pasadena, United States) and submitted to a second PCR round for indexing using the Nextera XT index primers 1 and 2. Thermocycling conditions consisted of a denaturation step of 4 min at 94°C, followed by 25 cycles of denaturation (15 s at 94°C), annealing (45 s at 56°C), and extension (60 s at 72°C), with a final elongation step (8 min at 72°C). These amplifications were performed on an EP Mastercycler Gradient System device (Eppendorf, Hamburg, Germany). The PCR products of approximately 650 nucleotides were run on 1% agarose gel electrophoresis, and the DNA fragments were plugged out and purified using a Wizard SV PCR purification kit (Promega Benelux, Netherlands). After purification, PCR products were quantified using the Quanti-IT PicoGreen (Thermo Fisher Scientific, United States) and were diluted to 10 ng/μL. A final quantification by quantitative (q)PCR of each sample in the library was performed using the KAPA SYBR^®^ FAST qPCR Kit (KapaBiosystems, United States) before normalization, pooling, and sequencing on a MiSeq sequencer using V3 reagents (Illumina, United States).

### Bioinformatics Analysis

The 16S rRNA gene sequence reads were processed with MOTHUR (Schloss et al., 2009). The quality of all sequence reads was denoised using the Pyronoise algorithm implemented in MOTHUR. The sequences were checked for the presence of chimeric amplification using ChimeraSlayer (developed by the Broad Institute^1^). The obtained read sets were compared to a reference data set of aligned sequences of the corresponding region derived from the SILVA database of full-length rRNA gene sequences^2^ implemented in MOTHUR (Pruesse et al., 2007; Quast et al., 2012). The final reads were clustered into OTUs using the nearest neighbor algorithm of MOTHUR with a 0.03 distance unit cut-off. A taxonomic identity was attributed to each OTU by comparison to the SILVA database using an 80% homogeneity cut-off. As MOTHUR is not dedicated to the taxonomic assignment beyond the genus level, all unique sequences for each OTU were compared to the SILVA dataset 111 using a BLASTN algorithm. For each OTU, a consensus-detailed taxonomic identification was given based on the identity (<1% mismatch with the aligned sequence) and the metadata associated with the best hit (validated bacterial species or not) (Delcenserie et al., 2014).

### 16S rDNA Data Analysis

A correcting factor for 16S rDNA gene copy numbers was applied for any taxon *i* (equation 1) (Kembel et al., 2012; Louca et al., 2018).

(1) $$A_{i}=N_{i}\times C_{i}$$

where A_i_ is the real abundance of 16S genes from that taxon; N_i_ is the number of reads for the taxon in the sample; and C_i_ is the genomic 16S copy number of that taxon. To obtain each gene copy number, Ribosomal RNA Database (rrnDB) (Stoddard et al., 2015) and EzBioCloud database (Yoon et al., 2017) were used.

Then, to compare the relative abundance of OTUs, the number of reads was normalized as described by Chaillou et al. (2015). The reads counts of each sample were divided by a sample-specific scaling factor (Fougy et al., 2016; Rouger et al., 2017):

(2) $$S_{i}=N_{i}/m_{e}$$

where S_i_ is the normalization factor associated with sample; N_i_ is the number of total reads in the sample I; m_e_ is the median value of total reads for all the samples of the dataset. Reads counts of all samples were then transformed into a percentage of each OTUs.

### Statistical Analysis

Mann–Withney test was used to compare the classical microbiology and ATPmetry results in relation with the cleaning treatments using the R software (R Core Team, 2008). All tests were considered significant when *p* ≤ 0.05. When non-colony was detected in the classical microbiology results, a value of half-limit of detection was used for the calculations (Hutchison et al., 2005; Ghafir et al., 2008).

The richness estimation (Chao1 estimator) and microbial biodiversity (inverse of the Simpson index, coverage index) were evaluated using MOTHUR (version 1.40.5)^3^ (Riquelme et al., 2015). The coverage index measures how well an environment was sampled and indicates the percentage of individuals sampled in a microbial community. The analysis is included with a coverage index of at least 0.9 (Lemos et al., 2011). Chao1 index estimates diversity from the abundance data (importance of rare OTUs). The inverse Simpson index reflects the effective number of species: the higher its value, the greater the diversity (with 1 as the lowest possible number).

Using the STAMP (v2+) software^4^, a two-sided Welch’s *t*-test was performed on metagenetic results, and confidence intervals were calculated according to the Newcombe–Wilson method. The differences were considered significant when *p* ≤ 0.05 (Parks et al., 2014).

## Results

### Ingredients

**FIGURE 2:**
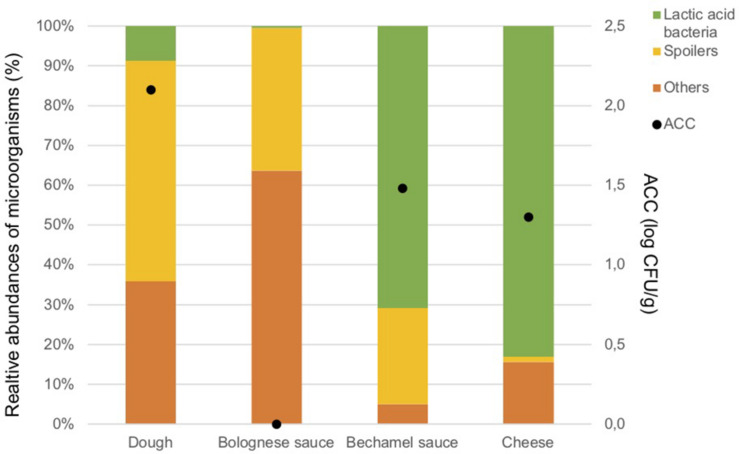
Aerobic Colony Counts (ACC) (log CFU/g) and relative abundances of the dominant bacterial groups (%) in ingredients.

The ingredients were analyzed once to evaluate the potential source of contamination in the food process. The results of ACC are in Figure 2. As expected, only a few microorganisms are present in the Bolognese and bechamel sauces due the cooking process. The dough, bechamel sauce, and cheese have low bacterial concentrations. The concentration of anaerobic colony was under the Dl in the ingredients.

Figure 2 illustrates also the results of the metagenetic analysis. To facilitate the interpretation of the graph, three groups of bacteria, namely “Spoilers,” “Lactic acid bacteria,” and “Others” were created based on the predominant groups identified. “Spoilers” include bacteria belonging to the *Enterobacteriaceae* family, *Pseudomonas* sp., *Leuconostoc* sp., and *Brochotrix thermosphacta*, as well as spore-forming bacteria which include *Bacillus* sp., *Geobacillus* sp., *Paenibacillus* sp., and *Anoxybacillus* sp. “Lactic acid bacteria” includes *Lactococcus* sp., *Lactobacillus* sp., and *Streptococcus* sp., whereas “Others” includes *Stenotrophomonas maltophilia* and all microorganisms that are present less than 1% in the analyzed sample and do not belong to the bacterial groups listed above.

Lactic acid bacteria are the most dominant in the cheese (*Lactococcus lactis*, 80.3%) and in the bechamel sauce (*Streptococcus* sp., 70.7%). A high number of taxa classified as “Others” are identified in the dough and Bolognese sauce (35.9 and 63.7%, respectively). Potential spoilers are present in the dough (55.4%), Bolognese sauce (35.9%), and bechamel sauce (24.2%).

Supplementary Material summarizes the ACC, metagenetic results, microbial richness, and diversity indicates of all samples collected in this study. All the ingredient samples have a coverage index above 0.9. The bechamel sauce has the highest value for the Chao1 index but the lowest value for the inverse Simpson index, indicating that many OTUs are related to the same bacterial species. Meanwhile, the Bolognese sauce has the lowest value for the Chao1 index but the highest value for the inverse Simpson, indicating that there are many bacterial species with few OTUs.

### Surfaces

Table 3 present the results of the ACC and ATPmetry for surface samples. The results indicate that some surfaces were highly contaminated during conventional cleaning, and significant decreases between conventional and enzymatic cleaning were observed for ACC (*p* = 0.042) and ATPmetry (*p* = 0.002).

**TABLE 3:** ACC (log CFU/cm^2^) and ATPmetry (pg cATP/ml) for processing surfaces in relation with cleaning treatment.

Cleaning treatment		ACC (log CFU/g)				ATPmetry (pg cATP/ml)			Group		Mann–Whitney Test - Conventional cleaning (Group A), Enzymatic cleaning (Group B)	
						
		n	P50	P90	Pmax	n	P50	P90	Pmax		ACC	ATPmetry
Conventional cleaning		8	2,0	7,7	8,3	17	0,6	39,7	929,2	A	*p* = 0.042	*p* = 0.002
Enzymatic cleaning	Reinforced enzymatic cleaning	18	1,3	2,7	3,2	5	0,2	0,5	0,7	B		
	Routine enzymatic cleaning 3x/wk	10	2,0	2,3	2,3	7	0,2	1,2	2,6	B		
	Routine enzymatic cleaning 2x/wk	7	DL	0,1	0,3	8	0,3	0,4	0,4	B		
	Routine enzymatic cleaning 1x/wk	4	<Dl			8	0,3	0,5	0,5	B		
Conventional cleaning		9	<Dl			8	0,2	0,5	0,9	A		
Enzymatic cleaning	Reinforced enzymatic cleaning 2 (OPC)	18	1,0	1,0	1,0	16	0,1	0,3	0,6	B		

*ACC, aerobic colony counts; Dl, detection limit; P50, percentile 50; P90, percentile 90; Pmax, percentile 100.*

**FIGURE 3:**
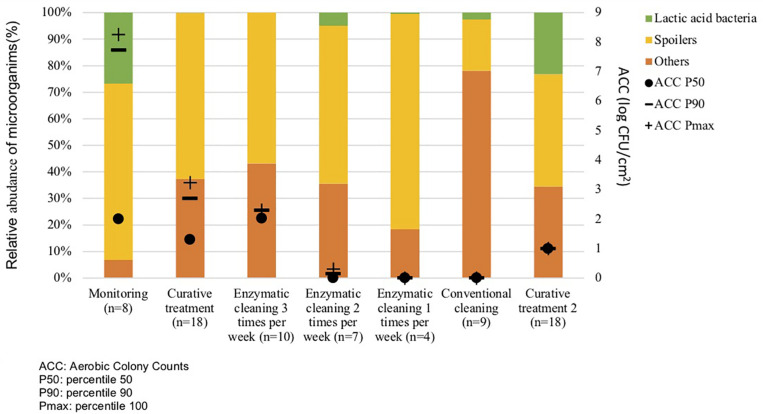
Percentiles 50, 90 and maximum of Aerobic Colony Counts (ACC) (log CFU/cm^2^) and mean abundances of the dominant bacterial groups (%) on processing surfaces in different cleaning treatments.

Metagenetic analyses indicate that the proportion of spoilers remained relatively stable on the surface during the different cleaning treatments (Figure 3 and Supplementary Material). However, the composition of microorganisms in this group changed with the cleaning method. *Pseudomonas* sp. (16.6 ± 32.5%) and *Enterobacteriaceae* (46.3 ± 60.8%) were the most abundant bacteria during the first conventional cleaning. Spore-forming bacteria were the most predominant group during curative enzymatic cleaning (49.1 ± 61.7%), routine enzymatic cleaning 3x/week (56.6 ± 75.7%), 2X/week (39.4 ± 48.5%), and 1x week (49.7 ± 49.5%). During the second conventional cleaning period, microbiological diversity increased, having a high proportion of “others” (78.12 ± 51.7%). Finally, when the second curative cleaning was reinstated, spore-forming bacteria reappeared at a high proportion (38.7 ± 61.7%). Figure F presents the results of the statistical analysis on the metagenetic data (Welsh t-test). Significant differences between conventional cleaning and enzymatic cleaning were noted on *Streptococcus* sp. (*p* < 0.029) and spore-forming bacteria (*p* < 0.046). These were more present during enzymatic cleaning. The implementation of the enzymatic cleaning on surfaces led to a decrease in bacterial concentration and a shift in bacterial composition. The coverage index for surface sampling was 0.986–0.993. Chao1 index values were relatively constant during the cleaning treatments. An increase in microbial diversity was observed after the implementation of the enzymatic cleaning with an increasing inverse Simpson index.

### Closed Circuits

Table 4 shows the results of classical microbiology analysis after the enzymatic treatment of closed circuits (closing pipes and tanks) during the CIP treatment. This treatment was carried once during the first reinforced enzymatic cleaning due to its practicality. No microorganisms and low ATP concentration were detected in the rinsing water before enzymatic cleaning. However, they both increased during enzymatic treatment in some part of the process, indicating a release of microorganisms and organic compounds during treatment.

**TABLE 4:** ACC (log CFU/ml) and ATPmetry (pg cATP/ml) for the closed circuits during enzymatic cleaning treatment.

Closed circuits	ACC	ATPmetry
	(log CFU/ml)	(pg cATP/ml)
Rinsing water	<Dl	0.6
Line 1	1.30	10748.5
Line 2	3.32	1230.5
Dough machine 1	3.23	735.9
Dough machine 2	7.48	1598.2
Bolognese cooking tank	<Dl	2.1
Bechamel cooking tank	<Dl	0.5

*ACC, aerobic colony counts; Dl, Detection Limit.*

**FIGURE 4:**
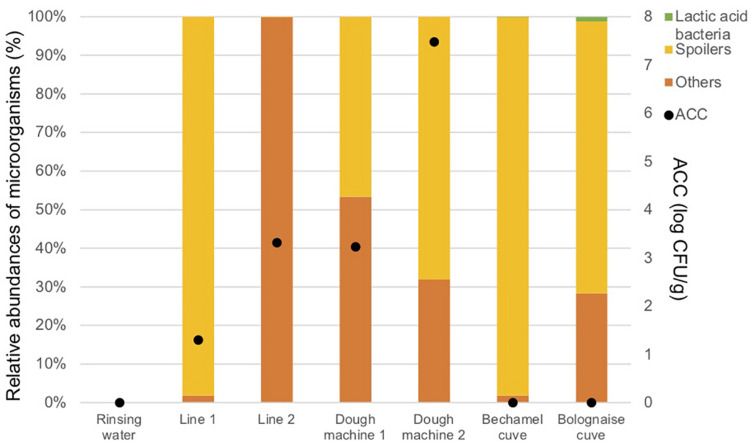
Aerobic Colony Counts (ACC) (log CFU/ml) and abundances of the dominant bacterial groups (%) in the closed circuits during the reinforced enzymatic treatments.

Figure 4 and Table 3 indicate that a high proportion of spoilers was released in line 1 (98.1%), which were mainly *Pseudomonas* and spore forming bacteria, in dough machine 1 (46.6%), mainly *Enterobacteriaceae*, and in dough machine 2 (68.1%), mainly *Enterobacteriaceae* and *Pseudomonas*. “Others” was predominant in line 2 (99.9%). Although no bacteria were detected by classical microbiology in the bechamel and Bolognese tanks, spoiling bacteria were detected in high proportions (98.0% and 70.5%), respectively. *Pseudomonas and Enterobacteriaceae* were mainly present in the Bolognese tank, whereas spore-forming bacteria were mainly detected in the bechamel tank. The coverage index values ranged from 0.941 to 0.995. The minimum values for Chao1 and inverse Simpson index were from line 2 and bechamel tank, having the presence of one dominant bacterial species, *Stenotrophomonas maltophilia* and *Brochotrix thermosphacta*, respectively. The highest values for Chao1 and inverse Simpson index were from the dough machine 2, which was also the sample with the highest concentration of total flora.

### Food Products

Table 5 lists the ACC and ANCC values in the final product at D_0_, D_end of the shelf life_, and D_end of the shelf life +7 days_ in relation to the cleaning method. As expected, the total flora increased during the shelf life. The P90 of ACC and ANCC decreased in food products analyzed at D_0_ by about 2 log UFC/g after the implementation of enzymatic cleaning, increased when conventional cleaning is reinstated, and decreased again after enzymatic cleaning. Significant differences for ACC were found between conventional and enzymatic cleaning for the finished products at D_0_ (*p* = 0.011), D_end of the shelf life_ (*p* = 0.029) and D_end of the shelf life +7 days_ (*p* = 0.027), and for ANCC at D_0_ (*p* = 0.010) and D_end of the shelf life +7 days_ (*p* = 0.004).

**TABLE 5:** ACC and ANCC of the food product at D_0_, D_end of the shelf life_, and D_end of the shelf life +7 days_ for different cleaning treatments.

**Cleaning treatment**			**ACC**									**ANCC**									**Group**	**Mann–Whitney Test − Conventional cleaning (Group A.) Enzymatic cleaning (Group B)**					
																							**ACC**			**ANCC**	
				**D_0_**			**D_end of the shelf life_**			**D_end of the shelf life + 7 days_**			**D_0_**			**D_end of the shelf life_**			**D_end of the shelf life + 7 days_**			**D_0_**	**D_end of the shelf life_**	**D_end of the shelf life + 7 days_**	**D_0_**	**D_end of the shelf life_**	**D_end of the shelf life + 7 days_**
											
		**n**	**P50**	**P90**	**Pmax**	**P50**	**P90**	**Pmax**	**P50**	**P90**	**Pmax**	**P50**	**P90**	**Pmax**	**P50**	**P90**	**Pmax**	**P50**	**P90**	**Pmax**							
Conventional cleaning		39	1,8	4,1	4,6	7,4	7,9	8,0	8,0	8,6	8,7	1,3	4,16	4,68	7,76	8,53	8,62	7,99	8,46	9,41	A	*p* = 0.011	*p* = 0.029	*p* = 0.027	*p* = 0.010	NS	*p* = 0.04
Enzymatic cleaning	Reinforced enzymatic cleaning (OPC + CIP)	12	1,7	2,2	2,3	8,4	9,1	9,2	7,8	8,1	8,2	<Dl	2,4	2,56	8,62	9,11	9,18	7,93	8,07	8,11	B						
	Routine enzymatic cleaning 3x/wk	13	1,4	1,7	1,7	6,6	7,9	8,0	7,1	8,8	8,9	1,48	1,48	1,48	6,66	7,79	8	7,36	8,78	8,89	B						
	Routine enzymatic cleaning 2x/wk	12	1,5	2,7	2,8	7,0	7,8	7,9	7,3	8,4	8,6	<Dl	2,65	2,81	7,79	8,16	8,2	7,35	8,08	8,2	B						
	Routine enzymatic cleaning 1x/wk	7	0,7	1,8	2,0	7,0	7,1	7,1	7,3	7,6	7,6	1	2,24	2,33	7,04	7,15	7,18	7,37	7,62	7,66	B						
Conventional cleaning 2		12	2,3	4,2	4,4	6,8	7,8	7,9	7,7	9,3	9,5	2,23	4,48	4,63	5,59	7,43	7,58	7,81	8,41	8,53	A						
Enzymatic cleaning	Reinforced enzymatic cleaning 2 (OPC)	9	1,6	1,9	1,9	5,5	6,0	6,1	5,3	6,0	6,1	2,2	2,82	2,9	5,51	9,16	9,26	4,26	8,54	8,63	B						

*NS, not significant; ACC, aerobic colony counts; ANCC, anaerobic colony counts; D_0_, food product analyzed at the beginning of the shelf life; D_*end of the shelf life*_, food product analyzed at the end of the shelf life; D_*end of the shelf +* 7 *days*_, food product analyzed 7 days after the end of the shelf life; Dl, detection limit; P50, percentile 50; P90, percentile 90; Pmax, percentile 100.*

**FIGURE 5:**
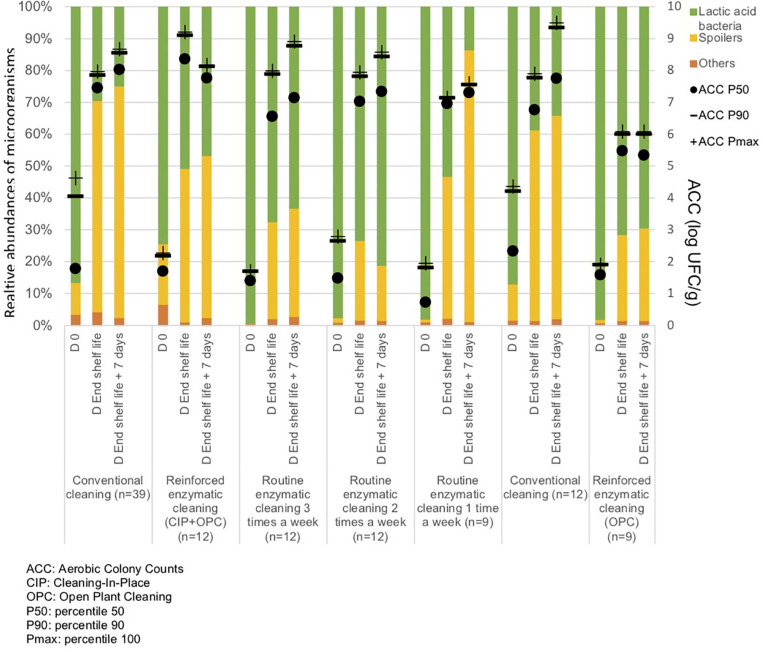
Percentiles 50, 90 and maximum of Aerobic Colony Counts (ACC) (log CFU/g) and mean abundances of the dominant bacterial groups (%) in the food products throughout their shelf life after different cleaning treatments.

**FIGURE 6:**
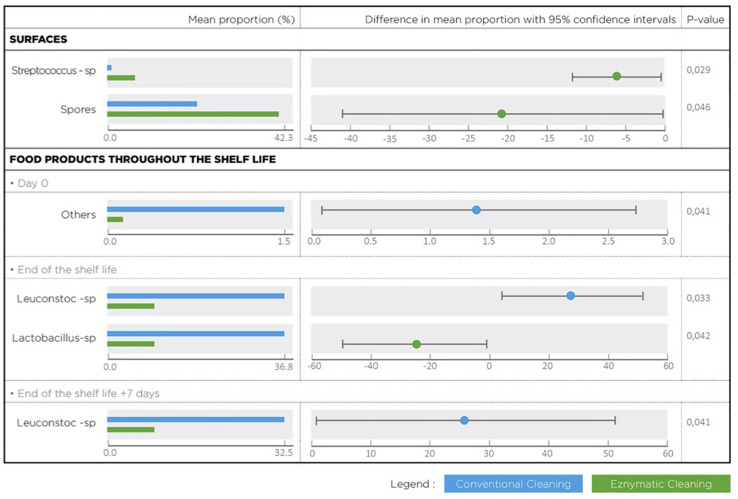
Bacterial species with a statistical difference between conventional and enzymatic cleanings of processing surfaces and in the lasagne throughout its shelf life.

From Figure 5 and Table 3, the main bacteria present at the start of shelf life were lactic acid bacteria (*Lactococcu*s sp.), regardless of the cleaning treatment. The proportion of spoilers decreased in the finished products at D_end of the shelf life +7 days_ after enzymatic cleaning, except when the enzymatic cleaning frequency was reduced to once a week. Figure 6 shows significant differences between conventional and enzymatic cleaning for the bacteria in the “Others” group in the product analyzed at D_0_, for *Leuconostoc* sp. and *Lactobacillus* sp. at D_end of the shelf life_, and *Leuconostoc* sp. at D_end of the shelf life +7 days_. The decrease in spoiling bacteria was compensated by the higher proportion of lactic acid bacteria. The coverage index ranged from 0.988–0.996 for the food products. Chao1 and the inverse Simpson index were relatively stable throughout the shelf life of the food products. The number of OTUs and the microbial diversity were constant and relatively lower compared to with the other samples.

## Discussion

Cleaning and disinfection are the major day-to-day controls for hard-surface vectors in food product contamination. If effective, they can reduce hazards within the processing environment (Holah, 2013). The cleaning process removes food residues, soils, and organic matters that accumulate on processing equipment and surfaces during production (Gabric et al., 2016). Microorganisms adhering on the food product’s contact surfaces could be an important source of potential contamination, thus leading to serious hygienic problems and economic losses due to food spoilage (Holah, 2013). Biofilms are the dominant lifestyle of bacteria and are also likely found within food industry premises. Bacteria that reside, accumulate, and persist in biofilms on surfaces with risks of subsequent transfer to food products are threats to food quality and safety (Galié et al., 2018). In the food industry, aggressive chemicals, such as sodium hydroxide and sodium hypochlorite, together with clean-in-place techniques are often used to mitigate undesirable biofilm effects. However, such approaches are not always effective for biofilm control (Meireles et al., 2016). Correct cleaning and disinfection strategies for biofilm eradication and prevention with documented effects under relevant conditions are necessary to overcome biofilms in food process industries (Simões et al., 2010).

Classical culture methods, such as agar plating, are not effective to detect biofilms due to the difficulty in culturing many biofilm bacteria, as well as the “viable but non-culturable” (VBNC) form with low metabolic activity. VBNC cells can be detected by using a culture-independent technique (Gião and Keevil, 2014). Developed in the last decades, next-generation sequencing methods have contributed immensely to the exploration of food microbiota (Galimberti et al., 2015) in beverages (Elizaquível et al., 2015; Pérez-Cataluña et al., 2018), vegetables (Lee et al., 2017; Gu et al., 2018), dairy (Nalbantoglu et al., 2014; Ceugniez et al., 2017; Porcellato et al., 2018), seafood (Parlapani et al., 2018; Silbande et al., 2018), and meat products (De Filippis et al., 2013; Benson et al., 2014; Greppi et al., 2015; Połka et al., 2015; Stoops et al., 2015; Zhao et al., 2015; Delhalle et al., 2016; Mann et al., 2016; Carrizosa et al., 2017; Cauchie et al., 2017; Kaur et al., 2017; Peruzy et al., 2019). However, only a few studies have investigated the microbial flora of food processing surfaces and equipment using next-generation sequencing methodologies (Bokulich and Mills, 2013; Bokulich et al., 2013; Mayo et al., 2014). The objective of this study was to evaluate the impact of enzymatic cleaning on the microbial flora of installations in a processed food industry, as well as in the final food product, using classical microbiology and metagenetic analysis. A food product with a very low initial bacterial flora concentration was selected to assess the change in the microbial flora from facilities to the final food product throughout its shelf life.

The interpretation of metagenetic analysis can sometimes be difficult when the number of identified microorganisms is high. To facilitate this, we summarized the results in the graphs by forming three groups of bacteria. “Spoilers” group contains microorganisms described as spoiling bacteria of various meat-based foodstuff (Iulietto et al., 2015; André et al., 2017). Lactic acid bacteria’s role is more ambiguous as they could be either be spoiling or protective, depending on the food product, environment, and species (Pothakos et al., 2015). Lastly, “Others” contained the bacterial taxa present at very low proportions (<1%) and were considered to have a minor impact on the microbial quality of the food product throughout the its shelf life (Cauchie et al., 2017).

Aerobic colony count values of the Bolognese and bechamel sauces were low due to the thermal process involved in the preparation of the ingredients. Lactic acid bacteria were dominant in the cheese and bechamel sauce. *Lactococcus lactis* is commonly used as a starter bacteria for cheese production (Fox and McSweeney, 2017). The milk used for the preparation of bechamel sauce contains an initial bacterial flora, which is eliminated in the cooking step. *Streptococcus sp.* is commonly found in raw milk (Quigley et al., 2013) and is heat-resistant; therefore, it could contaminate post-pasteurization (Flint et al., 2002). *Enterobacteriaceae* was identified in the Bolognese sauce and dough at a high proportion and is commonly present in red meat products (Doulgeraki et al., 2012) and wheat dough (Dinardo et al., 2019). *Pseudomonas* sp. was also present in the dough at a high proportion and has been identified in wheat dough previously (Celano et al., 2016; De Angelis et al., 2019; Menezes et al., 2020). Although the concentration of microorganisms in the ingredients was very low, they could be the starting point for contamination and lead to the spoilage of food products (ICMFS, 2006).

Some surfaces were highly contaminated during the first part of the study with conventional cleaning. These results are in accordance with other studies which evaluated the microbial concentration on food contact surfaces (Abdallah et al., 2014; Cunault et al., 2019). Because some equipment are difficult to clean, some organic residues could still be present even after cleaning and disinfection (da Costa Luciano et al., 2016; Gabric et al., 2016). The presence of organic residues promotes biofilm formation, which could be a permanent source of contamination (Wirtanen and Salo, 2016). The average concentration of AAC and ATP on surface decreased after the enzymatic cleaning. The implementation of enzymatic cleaning on surfaces resulted to a decrease in bacterial concentration and a shift in bacterial composition. *Pseudomonas* sp. and *Enterobacteriaceae* were mainly present during conventional cleaning, and they are described as biofilm formers and potential spoilers (López et al., 2010). The increasing proportion of spore-forming bacteria during enzymatic cleaning could be attributed to their higher resistance against the stress brought by the sanitizing process (Maillard, 2016). *Brochotrix thermosphacta* and *Enterobacteriaceae* have disappeared from surfaces when enzymatic cleaning was done three times per week.

Although the closed circuits were treated with conventional cleaning prior to enzymatic cleaning, there is a significant release in microorganisms and ATP in some parts of the installations during the treatment, especially for lines 1 and 2 and dough machines 1 and 2. No bacteria was detected by classical microbiology in the Bolognese and bechamel tanks due to the thermal inactivation of microorganisms during the cooking process (ICMFS, 2006). Bacteria identified in the dough machine and Bolognese and bechamel tanks are related to the ingredients used in these equipment’s. Several studies have described that conventional sanitizing process does not completely eliminate the microbial flora in closing pipes (Lelièvre et al., 2002; Bremer et al., 2006; Kumari and Sarkar, 2014, 2016; Liu et al., 2014). For example, Parijs and Steeenackers. have shown that microbial contamination after the CIP process in several breweries was reduced by less than 75% in 52% of the samples and was even increased in 24% of the samples, indicating that CIP is insufficient, and improving antimicrobial treatments is essential (Parijs and Steenackers, 2018). A high proportion of *Pseudomonas* sp. was found for the line 1 (67,9%) and to a lesser extent in other equipment as the dough machine 2 (36, 8%) and the Bolognese tank (37,6%). *Pseudomonas* spp. is among the bacteria most frequently isolated from surfaces in the food industry and it produces multispecies biofilm on the wall of tanks and pipelines before heat processing (Shirtliff et al., 2002; Marchand et al., 2012). Spore-forming bacteria were detected in the bechamel tank at a high proportion (97,9%). Several previous studies assessed CIP procedures to eliminate spore-forming bacteria and demonstrated that the efficacy is related to the several parameters such as the surface chemistry, shear forces and the detergent applied during the cleaning (Lelièvre et al., 2002; Sundberg et al., 2011; Faille et al., 2013). Sporulation could occur in biofilms, suggesting that biofilms would be a significant source of food contamination with spores (Wijman et al., 2007; Faille et al., 2014).

Expectedly, ACC results in the food products showed an increasing concentration of the bacterial flora during shelf life. After the implementation of enzymatic cleaning, the bacterial flora at the beginning of the shelf life decreased to 2 log CFU/g. The hygiene of food installations has a measurable effect on the food product, especially when the initial concentration of the food products is very low (ICMFS, 2006). The products at D_0_ were mainly composed of *Lactococcus* sp., which was mainly present in the cheese used as topping of the lasagne. Spoiling bacteria, such as *Enterobacteriaceae sp.* and *Leuconostoc* sp., were predominant in the food product along its shelf life during conventional cleaning. After the implementation of enzymatic cleaning, the proportion of spoiling bacteria decreased in favor of lactic acid bacteria, such as *Lactobacillus* sp. and *Lactococcus* sp., during the shelf life of the products. However, when the frequency of enzymatic cleaning was reduced to once a week, the proportion of spoiling bacteria increased again. Therefore, a minimum frequency of enzymatic cleaning is necessary to maintain a low proportion of spoiling bacteria in food products. Although there was a significant decrease in *Leuconostoc* sp. in favor of *Lactobacillus sp.*, it was not possible to affirm that the food product was not spoiled. Complementary tests, such as sensory analyses, are necessary to confirm that the product maintains all its microbial and technical qualities throughout the shelf life.

Several studies in meat microbiology have established that spoilage is caused by only a fraction of the initial microbial flora that dominates the food product throughout its shelf life (Nychas et al., 2008; Doulgeraki et al., 2012). These spoilage microorganisms have been designated as E(S)SOs due to their ability to eventually become dominant in the spoilage flora (Nychas et al., 2008; Pennacchia et al., 2011). *Leuconostoc* sp. was described as a spoilage bacteria in several food products, such as ready-to-eat food products (Petruzzi et al., 2017; Pellissery et al., 2020). This bacterium can grow very quickly and dominate the bacterial flora of food products packaged in modified atmosphere, even if its initial concentration is very low (Doulgeraki et al., 2012). During conventional cleaning, it was present in the food product at a very low proportion at the beginning of the shelf life and became the most predominant bacteria at the end of the shelf life. After enzymatic cleaning, its proportion was reduced in the food products along the shelf life. It was also detected on surfaces at a very low proportion, regardless of the cleaning treatment. However, ACC showed that bacterial concentration on surfaces was reduced during enzymatic treatment, including that of *Leuconostoc* sp. It is therefore likely that the decrease in the concentration of *Leuconostoc* sp. in the food installations leads to its reduction in the final food product.

Few studies are available comparing different cleaning methods on the microbial ecology in agro-industrial facilities and no studies have used high-throughput sequencing methods to describe the microbial population of industrial facilities and finished products according to different types of cleaning. High throughput sequencing coupled with classical microbiology methods provides useful information on the dynamics of bacterial populations according to environmental conditions, such as the change of cleaning methods. This study was carried out in a processing industry which produces the same product along the time and where the bacterial concentration in the food product is low at the beginning of its shelf life. In those conditions, it is possible to observe changes of the microbial flora in the food products following the change of cleaning procedures of the installations, which would probably be more difficult to observe in foodstuffs with more variable and higher bacterial flora concentration such as perishable foods. Future studies could also be conducted to evaluate cleaning methods on the microbial flora of instillations and the impact on finished products in other food sectors such as dairies, meat or fish industries.

A decrease in the microbial flora concentration and the proportion of spoilage bacteria on installations surfaces was observed after enzymatic cleaning compared to conventional cleaning. At the same time, a reduction of the initial concentration at the beginning of the shelf life and a reduction of the spoiling bacteria throughout the shelf life of the food product was also observed. Enzymic cleaning has led to an improvement in the hygiene of the facilities and the microbial quality of the food throughout the shelf life. Although its effectiveness in removing organic residues and biofilms is increasingly studied, this type of cleaning is not yet commonly used in the food industry. Depending on the installations, production method, and the food product itself with its specific risks, enzymatic cleaning should be considered in combination with conventional sanitizing methods to improve plant hygiene.

## Data Availability Statement

The datasets presented in this study can be found in online repositories. The names of the repository/repositories and accession number(s) can be found at: https://www.ncbi.nlm.nih.gov/, PRJNA615390.

## Author Contributions

LD contributed to design and followed up the study. BT performed the statistical analysis on metagenetic results. SF and MB contributed to technical support for enzymatic cleaners. AF carried out the bioinformatics on metagenetic analysis. SB carried out the metagenetic analysis. GD supervised the study. All authors contributed to the article and approved the submitted version.

## Conflict of Interest

MB and SF were employed by the Realco. SB and AF were employed by the Genalyse Partner. The remaining authors declare that the research was conducted in the absence of any commercial or financial relationships that could be construed as a potential conflict of interest.

## Footnotes

1 http://microbiomeutil.sourceforge.net/#A_CS

2 http://www.arb-silva.de/, version v1.2.11.

3 http://www.mothur.org

4 https://beikolab.cs.dal.ca/software/STAMP

## Abbreviations

ACC: aerobic colony count
ANCC: anaerobic colony counts
ATP: adenosine triphosphate
CIP: cleaning-in-place
Dl: detection limit
ESSOs: ephemeral/specific spoilage organisms
OPC: open plant cleaning
OTU: operational taxonomic unit
P50: percentile 50
P90: percentile 90
Pmax: percentile 100
qPCR: quantitative polymerase chain reaction.
